# Clinical genetics of spondylocostal dysostosis: A mini review

**DOI:** 10.3389/fgene.2022.996364

**Published:** 2022-11-25

**Authors:** Muhammad Umair, Muhammad Younus, Sarfraz Shafiq, Anam Nayab, Majid Alfadhel

**Affiliations:** ^1^ Medical Genomics Research Department, Ministry of National Guard Health Affairs (MNGH), King Abdullah International Medical Research Center (KAIMRC), King Saud Bin Abdulaziz University for Health Sciences, Riyadh, Saudi Arabia; ^2^ State Key Laboratory of Membrane Biology and Beijing Key Laboratory of Cardiometabolic Molecular Medicine, Institute of Molecular Medicine, College of Future Technology and Peking-Tsinghua Center for Life Sciences and PKU-IDG/McGovern Institute for Brain Research, Peking University, Beijing, China; ^3^ Department of Anatomy and Cell Biology, University of Western Ontario, London, ON, Canada; ^4^ Department of Biotechnology, Fatima Jinnah Women University, Rawalpindi, Pakistan; ^5^ Genetics and Precision Medicine Department, King Abdullah Specialized Children Hospital (KASCH), King Abdulaziz Medical City, Ministry of National Guard Health Affairs (MNG-HA), Riyadh, Saudi Arabia

**Keywords:** Spondylocostal dysostosis, SCDO, Genetic skeletal disorders, SCDO1-7, Notch-signaling pathway

## Abstract

Spondylocostal dysostosis is a genetic defect associated with severe rib and vertebrae malformations. In recent years, extensive clinical and molecular diagnosis advancements enabled us to identify disease-causing variants in different genes for such severe conditions. The identification of novel candidate genes enabled us to understand the developmental biology and molecular and cellular mechanisms involved in the etiology of these rare diseases. Here, we discuss the clinical and molecular targets associated with spondylocostal dysostosis, including clinical evaluation, genes, and pathways involved. This review might help us understand the basics of such a severe disorder, which might help in proper clinical characterization and help in future therapeutic strategies.

## 1 Introduction

The spondylocostal dysostosis (SCDO), a subclass of a Jarcho–Levin syndrome, represents a rare heritable group of genetic disorders. SCDO is characterized by congenital defective vertebral segmentation and rib deformation due to imperfect alignments, fusion, or reduction in numbers. The patient often presents with a short trunk and scoliosis (Solomon et al., 1978; Mortier et al., 1996; Gucev et al., 2010). SCDO can be characterized as familial or sporadic because of its autosomal recessive and dominant inheritance pattern (Turnpenny et al., 1993). SCDO is generally inherited as an autosomal recessive trait due to mutations of the genes that are involved in the Notch-signaling pathway such as Delta-like canonical Notch ligand 3 (*DLL3*) (Bulman et al., 2000), mesoderm posterior protein 2 (*MESP2*) (Whittock et al., 2004a; Whittock et al., 2004b), *LFNG* (Sparrow et al., 2006), and *HES7* genes (Sparrow et al., 2008). Patients with familial SCDO clinically present with the most common symptoms such as anal and urogenital anomalies, congenital heart disease, limb abnormalities, plagiocephaly-torticollis deformity sequence, diaphragmatic hernia, and neuronal tube defects (in males) (Cetinkaya et al., 2008), while patients with sporadic SCDO present with a wide variety of symptoms such as asymmetrical thoracic region and scoliosis (Mortier et al., 1996). Numerous sporadic SCDO cases have been reported with VATER, VACTERL, and MURCS associations along with DiGeorge, Alagille, and Robinow syndromes. Associations with an intellectual disability and other abnormal neurological syndromes are unusual (Turnpenny et al., 2009; Turnpenny et al., 2017).

In the present review, we have classified spondylocostal dysostosis based on gene identification and associated clinical phenotypes. A total of 185 and 284 entries were obtained using the mesh “spondylocostal dysostosis” in the OMIM and PubMed (NCBI). They include both syndromic and non-syndromic spondylocostal dysostosis. Autosomal recessive spondylocostal dysostoses have been classified into seven types and are presented in Table 1, which might be helpful for researchers and clinicians to have a quick overview of the disorder, help in molecular diagnosis, and further management plans.

**TABLE 1:** Spondylocostal dysostosis-associated genes, associated clinical representation, and function in the Notch-signaling pathway.

S.No	Gene	Disease	Clinical features observed	Function	Reference
1	*DLL3* (OMIM 602768)	Spondylocostal dysostosis 1 (OMIM 277300)	Dwarfism, respiratory infection, short trunk, rib anomalies, and vertebral fusion	Encodes Delta proteins which functions as Notch ligands and inhibits signaling	Kusumi et al. (2004)
2	*MESP2* (OMIM 605195)	Spondylocostal dysostosis 2 (OMIM 608681)	Disproportionate short stature, fusion of ribs at costovertebral junction, angular vertebrae, vertebral clefts, and sickle-shaped vertebrae	Encodes proteins, basically transcription factors that belong to the bHLH family. It plays an important role in the somite formation *via* interactions with Notch-signaling pathways	Whittock et al. (2004b)
3	*LFNG* (OMIM 602576)	Spondylocostal dysostosis 3 (OMIM 609813)	Short stature, fused ribs, multiple vertebral anomalies, kyphosis, and scoliosis	Encodes a glycosyltransferase, that is, involved in Notch1 receptor signaling *via* post-translational modification of Notch receptors	Sparrow et al. (2006)
4	*HES7* (OMIM 608059)	Spondylocostal dysostosis 4 (OMIM 613686)	Short stature, dextrocardia, cardiovascular anomalies, restrictive ventilatory defect, ribs anomalies, and fusion	Encodes a transcriptional repressor protein which helps in accurate modeling of axial skeleton. It belongs to the hairy and enhancer of split family of bHLH transcription factors. This gene is regulated by Notch signaling	Sparrow et al. (2008)
5	*TBX6* (OMIM 602427)	Spondylocostal dysostosis 5	Disproportionate short stature, fusion of ribs, ribs anomalies, extra or missing ribs, scoliosis, and butterfly vertebrae	Encodes a T-box transcription factor which helps in activating the *MESP2* and *DLL1* gene expression that are key to the regulation of developmental processes	Wu et al. (2015)
				It belongs to a conserved gene family that has a common T-box and a DNA-binding domain	
6	*RIPPLY2* (OMIM 609891)	Spondylocostal dysostosis 6 (OMIM 616566)	Spinal canal stenosis, absence of posterior elements of upper cervical vertebrae, hemi-vertebrae in cervical and thoracic spine, cervical kyphosis, thoracic scoliosis, and spinal cord compression	It encodes a nuclear protein essential for the vertebrate somitogenesis. It acts as a transcriptional repressor protein by interacting with the transcriptional repressor Groucho and a carboxy-terminal RIPPLY homology domain *via* a tetrapeptide WRPW motif. Mutant mice models displays defective somitogenesis	McInerney-Leo et al. (2015)
7	*DLL1* (OMIM 606582)	Spondylocostal dysostosis 7	Kyphosis, scoliosis, delayed development, cortical dysplasia, mall cerebellum, and autistic features	Encodes *DLL1* which is a human homolog of the Notch Delta ligand and has an important role in mediating cell fate decisions during hematopoiesis. Its related pathways involve signaling by Notch1 and Notch2 activation and transmission of signal to the nucleus	Barhoumi et al. (2019)

### 1.1 Clinical description

Patients with SCDO have short trunks relative to their height, a short neck, and often present with thoracic insufficiency and mild to severe non-progressive scoliosis. Neonates often clinically present with mild to moderate respiratory insufficiency due to a smaller thorax. Neonatal lung growth may improve by the age of 2 years, which supports their normal growth and development. Such patients can survive but are at a higher risk of life-threatening complications such as pulmonary hypertension. Male patients with SCDO may be at a higher risk of inguinal hernia (Turnpenny et al., 2017).

### 1.2 Classification

SCDO is classified into seven types based on pathogenic mutations in different genes (Table 1). Autosomal recessive SCDO is isolated in nature, and is confined to the vertebrae and ribs. However, additional syndromic types have been reported in some of the subtypes.

### 1.3 Spondylocostal dysostosis type 1 (SCDO1; *DLL3*)

SCDO1 is caused by pathogenic biallelic mutations in the Delta-like canonical Notch ligand 3 (*DLL3*) gene (MIM 602768) located on chromosome 19q13.2 with autosomal recessive mode of inheritance. The clinical features, reported in the affected individuals having *DLL3* mutations, are the irregularity of the vertebral column on spinal radiographs in early childhood and the prenatal stage. “Pebble beach sign” of each vertebral body in the fetus is a characteristic feature, which is observed having an ovoid or round shape with smooth boundaries (Turnpenny et al., 2003). As a result, the fetus develops the pebble beach appearance of the vertebrae and gives rise to multiple irregularities to the vertebral column. In such a situation, an MRI is more feasible to view the irregularities as compared to X-rays.

The *DLL3* gene (NM_016941.4) consists of eight exons encoding a 618-amino acid (NP_058637.1) long protein. The long encoded *DLL3* protein is composed of different domains such as a Delta–Serrate-Lag2 region (DSL), six epidermal growth factor-like domains (EGF), and a transmembrane (TM) domain. *DLL3* has been shown to be involved in the somite boundary formation and cell-signaling mechanism as it has shown a spatially restricted expression pattern when studied in different animal models (Dunwoodie et al., 2002; Kusumi et al., 2004; Dunwoodie S. L., 2009). To date, only 31 disease-causing mutations have been reported in the *DLL3* (HGMD, 2021). Out of these, 28 homozygous mutations have been identified in the *DLL3* gene associated with SCDO1. These mutations included seven nonsense mutations, six missense mutations, ten small deletions, and five small insertions (Table 2).

**TABLE 2:** Mutations reported to date in genes associated with spondylocostal dysostosis (HGMD^®^ Professional 2022.2).

Number of mutation	Gene name	cDNA	Protein	Phenotype	Types of mutation
1	*DLL3*	c.150C>A	p.C50*	Spondylocostal dysostosis	Nonsense
2	*DLL3*	c.621C>A	p.C207*	Spondylocostal dysostosis	Nonsense
3	*DLL3*	c.674G>A	p.S225N	Congenital scoliosis	Missense
4	*DLL3*	c.712C>T	p.R238*	Spondylocostal dysostosis	Nonsense
5	*DLL3*	c.805G>A	p.G269R	Vertebral malformation	Missense
6	*DLL3*	c.810C>A	p.C270*	Spondylocostal dysostosis	Nonsense
7	*DLL3*	c.926G>A	p.C309Y	Spondylocostal dysostosis	Missense
8	*DLL3*	c.1086C>A	p.C362*	Spondylocostal dysostosis	Nonsense
9	*DLL3*	c.1138C>T	p.R380C	Spondylocostal dysostosis	Missense
10	*DLL3*	c.1154G>A	p.G385D	Spondylocostal dysostosis	Missense
11	*DLL3*	c.1164C>A	p.C388*	Spondylocostal dysostosis	Nonsense
12	*DLL3*	c.1511G>A	p.G504D	Spondylocostal dysostosis	Missense
13	*DLL3*	c.329delT	p.(Val110Glyfs*22)	Spondylocostal dysostosis	Small deletion
14	*DLL3*	c.395delG	p.(Gly132Glufs*109)	Spondylocostal dysostosis	Small deletion
15	*DLL3*	c.602delG	p.(Gly201Valfs*40)	Spondylocostal dysostosis	Small deletion
16	*DLL3*	c.618delC	p.(Cys207Alafs*34)	Spondylocostal dysostosis	Small deletion
17	*DLL3*	c.868_870+8del11	p.?	Spondylocostal dysostosis	Small deletion
18	*DLL3*	c.945_946delAT	p.(Ala317Argfs*17)	Spondylocostal dysostosis	Small deletion
19	*DLL3*	c.948_949delTG	p.(Ala317Argfs*17)	Spondylocostal dysostosis	Small deletion
20	*DLL3*	c.1365_1381del17	p.(Cys455Trpfs*5)	Spondylocostal dysostosis	Small deletion
21	*DLL3*	c.1418delC	p.(Ala473Glufs*75)	Spondylocostal dysostosis	Small deletion
22	*DLL3*	c.1440delG	p.(Pro481Argfs*67)	Spondylocostal dysostosis	Small deletion
23	*DLL3*	c.599_603dupGCGGT	p.(Pro202Alafs*41)	Spondylocostal dysostosis	Small insertion
24	*DLL3*	c.602_614dup13	p.(Pro206Serfs*14)	Spondylocostal dysostosis	Small insertion
25	*DLL3*	c.1183_1184insCGCTGC	p.(Cys395delinsSerLeuArg)	Spondylocostal dysostosis	Small insertion
26	*DLL3*	c.1238_1255dup18	p.(His413_Ala418dup)	Spondylocostal dysostosis	Small insertion
27	*DLL3*	c.1291_1307dup17	p.(Pro437Thrfs*117)	Spondylocostal dysostosis	Small insertion
28	*DLL3*	c.661C>T	p.R221*	Hemi-vertebrae and rib fusion	Nonsense
1	*MESP2*	c.307G>T	p.E103*	Spondylocostal dysostosis	Nonsense
2	*MESP2*	c.367G>T	p.E123*	Spondylocostal dysostosis	Nonsense
3	*MESP2*	c.373C>G	p.L125V	Spondylocostal dysostosis	Missense
4	*MESP2*	c.688C>T	p.Q230*	Spondylocostal dysostosis	Nonsense
5	*MESP2*	c.737G>A	p.W246*	Spondylocostal dysostosis	Nonsense
6	*MESP2*	c.1166A>G	p.E389G	Spondylocostal dysostosis	Missense
7	*MESP2*	c.599delA	p.(Gln200Argfs*281)	Spondylocostal dysostosis	Small deletion
8	*MESP2*	c.180_193dup14	p.(Glu65Alafs*60)	Spondylocostal dysostosis	Small insertion
9	*MESP2*	c.500_503dupACCG	p.(Gly169Profs*199)	Spondylocostal dysostosis	Small insertion
1	*LFNG*	c.446C>T	p.T149I	Spondylocostal dysostosis	Missense
2	*LFNG*	c.564C>A	p.F188L	Spondylocostal dysostosis	Missense
3	*LFNG*	c.583T>C	p.W195R	Spondylocostal dysostosis	Missense
4	*LFNG*	c.601G>A	p.D201N	Spondylocostal dysostosis	Missense
5	*LFNG*	c.761C>T	p.T254M	Spondylocostal dysostosis	Missense
6	*LFNG*	c.842C>A	p.T281K	Spondylocostal dysostosis	Missense
7	*LFNG*	c.139_142delGGCC	p.(Gly47Profs*97)	Autism spectrum disorder	Small deletion
8	*LFNG*	c.372delG	p.(Lys124Asnfs*21)	Spondylocostal dysostosis	Small deletion
9	*LFNG*	c.44dupG	p.(Ala16Argfs*135)	Spondylocostal dysostosis	Small insertions
1	*HES7*	c.73C>T	p.R25W	Spondylocostal dysostosis	Missense
2	*HES7*	c.86A>G	p.N29S	Spondylocostal dysostosis	Missense
3	*HES7*	c.172A>G	p.I58V	Spondylocostal dysostosis	Missense
4	*HES7*	c.556G>T	p.D186Y	Spondylocostal dysostosis	Missense
5	*HES7*	c.385_394dup10	p.(Arg132Glnfs*42)	Spondylocostal dysostosis	Small insertion
1	*TBX6*	c.422T>C	p.L141P	Spondylocostal dysostosis	Missense
2	*TBX6*	c.449G>A	p. p.R150H	Spondylocostal dysostosis	Missense
3	*TBX6*	c.661C>A	p.H221N	Spondylocostal dysostosis	Missense
4	*TBX6*	c.699G>C	p.W233C	Spondylocostal dysostosis	Missense
5	*TBX6*	c.1148C>A	p.S383*	Spondylocostal dysostosis	Nonsense
6	*TBX6*	c.994delG	p.(Glu332Lysfs*166)	Spondylocostal dysostosis	Small deletion
1	*RIPPLY2*	c.238A>T	p.R80*	Vertebral segmentation defects	Nonsense
2	*RIPPLY2*	c.240–4T>G		Vertebral segmentation defects	Splice site
1	*DLL1*	c.1534G>A	p.G512R	Vertebral malformation	Missense

### 1.4 Spondylocostal dysostosis type 2 (SCDO2; *MESP2*)

SCDO2 is reported to be inherited as an autosomal recessive disorder, and is caused due to the mutation in the *MESP2* gene which is located on chromosome 15q26.1. The mutation in mesoderm posterior basic helix–loop–helix transcription factor 2 **(** *MESP2*) (MIM 605195) may be a homozygous or a compound heterozygous mutation. SCDO2 reported cases that exhibit clinical features such as truncal shortening, short necks, and spondylocostal dysostosis. As compared to SCDO1 (*DLL3*), the affected individuals have relatively mildly affected lumbar vertebrae as compared to the thoracic region (Whittock et al., 2004b).

In an Arab-Lebanese family, reported by Whittock et al. (2004a), two affected individuals exhibited spondylocostal dysostosis features, such as short necks and truncal shortening without any other abnormalities. In the same family, a biallelic 4-bp duplication (c.500_503dup) has been identified in the *MESP2* (NM_001039958.2, NP_001035047.1) gene as the main cause of the disease. It has been found that the mutation occurs in a sequence following the basic helix–loop–helix (bHLH) domain which leads to a frameshift, hence resulting in premature truncation (Whittock et al., 2004b).

**FIGURE 1:**
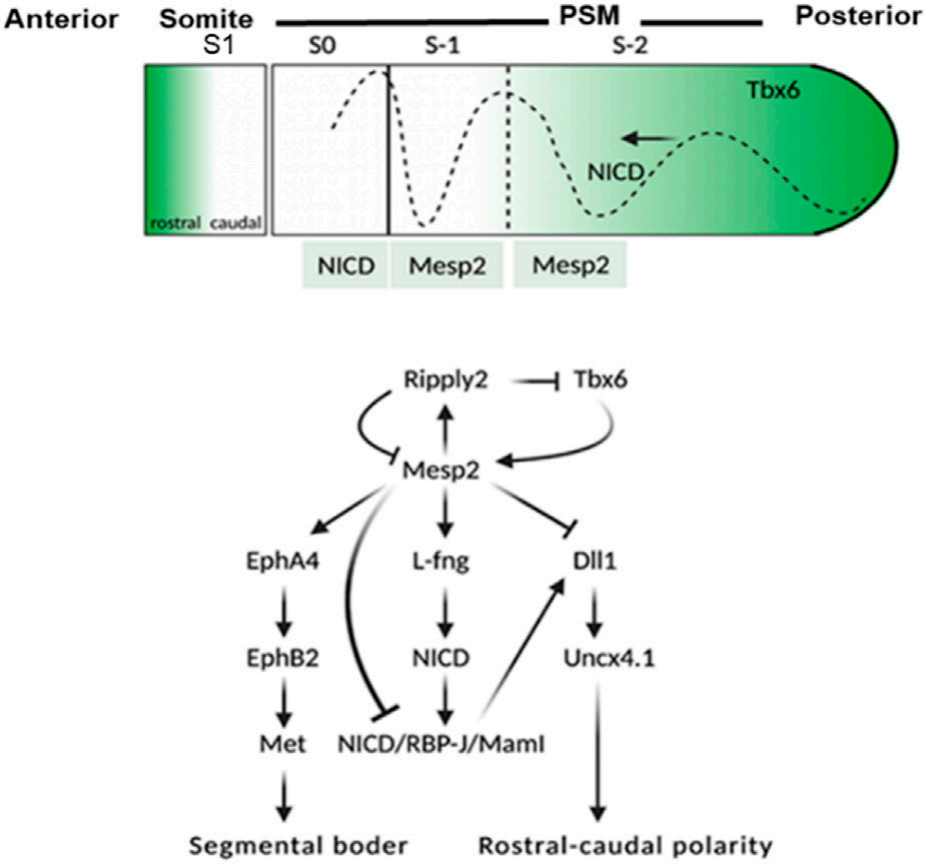
Schematic representation of events involved in the activation of *MESP2*. *TBX6* along with Notch1 signaling leads to the expression of *MESP2* in the anterior presomitic mesoderm (NICD dotted line). The dotted line toward the anterior along with the black arrow shows the Notch1 signal wavers in the presomitic mesoderm. As *MESP2* protein is formed within S1, NICD is suppressed indirectly *via* activation of *LFNG*, and directly *via* inhibition of *DLL1*. The expression of *MESP2* protein inhibits the expression of *DLL1* and *Uncx4.1* caudal genes; however, it induces the expression of rostral genes EphA4, EphB2, and followed by mesenchymal-to-epithelial transition (MET) within somites leading to segmentation of the rostral-caudal polarity within the somites is generated *via* a *RIPPLY2*–*MESP2* negative feedback loop. S1 = formed somite; S0 = somite under formation; S1 and S2 = somite primordia.

*MESP2* (MIM 605195; NM_001039958.2) consists of two exons (1614 bp) and encodes a protein comprising 397 amino acids (NP_001035047.1). The full-length *MESP2* protein consists of an N-terminal domain succeeded by a basic helix–loop–helix (bHLH) domain, a GQ (glycine and glutamine repeats)-rich region, and finally, a C-terminal domain. *MESP2* belongs to a family of transcriptional regulatory proteins that have a core helix–loop–helix (bHLH) domain, and plays a key role in rostro-caudal polarization and somite formation (Figure 1) (Mitsuru Morimoto et al., 2005) (Morimoto et al., 2007). To date, only nine mutations have been reported in the *MESP2* gene and only nine have been associated with SCDO2, which include four nonsense mutations, two missense mutations, one small deletion, and two small insertions (Table 2).

*MESP2* is integral to somite boundary formation, and is important for the development of anterior somatic compartment *via* suppression of Notch signaling (Figure 1). *MESP2* expression was analyzed by Cornier et al. (2008) and Saga et al. (1997) in mouse models. They argued that *MESP2* expressed as stripe in the presomitic mesoderm which is indicative of the future somite. It is these stripes that form a support for the vertebral development in the spine of adult mice. *MESP2* expression is localized in the anterior somatic compartment. *MESP2* gene knockout mice show severe vertebral segmentation defects along with proximal rib fusion which is parallel to that observed in STD patients.

### 1.5 Spondylocostal dysostosis type 3 (SCDO3; *LFNG*)

SCDO3 is clinically characterized by having long slender fingers, camptodactyly, and congenital vertebral anomalies. Radiological scans have revealed multiple vertebral ossification centers in the thoracic region of the vertebral column (Sparrow et al., 2006). SCDO3-carrying individuals exhibit more severe spine shortening as compared to SCDO1 and SCDO2 individuals, although ribs deformities have been reported to be similar to SCDO1 and SCDO2 cases. In 2006, Sparrow *et al* reported a biallelic missense mutation (c.564C>A; p.F188L) in the Leng O-fucosylpeptide 3-beta-N-acetylglucosaminyltransferase (*LFNG*) gene located on chromosome 7p22.3 as a candidate gene in a Lebanese proband. Recently, a proband from a Japanese family has been reported having features of severe multiple vertebral deformities starting from the cervical to sacral spine. Novel compound heterozygous variants [c.372delG (p.K124Nfs*) and c.601G>A (p.D201N)] in the *LFNG* gene have been identified which further validates the role of this gene in causing spondylocostal dysostosis type 3 in humans (Otomo et al., 2019).

*LFNG* (NM_001040167.2) is composed of a total of eight exons (2377bp) located on chromosome 7p22.3, encoding a 379-aa long protein (NP_001035257.1) To date, fourteen mutations have been reported in the *LFNG* gene responsible for causing SCDO3 phenotypes (HGMD; 2018). To date, only 14 mutations have been reported in the *LFNG* gene and only nine have been associated with SCDO3, including two small deletions, one small insertion, and six missense variants (Table 2). The other phenotypes reported include autism spectrum disorder (ASD), scoliosis, Asperger’s syndrome, and tetralogy of Fallot.

Apart from vertebral defects, the proband reported by Sparrow et al. (2006) had camptodactyly of the left index finger. The authors suggested that these features might not be due to the *LFNG* mutation, since according to one study, lack of *LFNG* expression in mouse models did not show limb defects (Zhang et al., 2002). It has been shown that the *LFNG* expression is restricted to the hemangioblasts (precursors of blood vessels) in a developing vertebrate limb. However, the expression of *LFNG* in the human embryo is still elusive, and human limb development might be different from that in the mouse (Sparrow et al., 2006).

### 1.6 Spondylocostal dysostosis type 4 (SCDO4; *HES7*)

SCDO4 is a severe autosomal recessive disorder triggered by the *HES7* gene mutation located on chromosome 17p13.1. A proband in a Caucasian Mediterranean origin consanguineous family had features such as lumbosacral myelomeningocele; hydrocephalus; myelomeningocele; bell-shaped, shortened thorax; stenotic anus; and talipes. The radiological examination of the proband also revealed the shortening of the spine and contiguous and multiple vertebral defects. The parents were normal, second cousins, having two healthy kids (Sparrow et al., 2008). Molecular analysis of the proband led to the identification of a homozygous missense mutation (c.73C>T; p. Arg25Trp) in the *HES7* gene.

Later, two affected cases from a non-consanguineous Italian family were reported to have spondylocostal dysostosis. The affected individuals showed multiple anomalies, multiple rib fusions with 11 pairs of bilateral ribs, short trunk, and short stature. MRI results revealed multiple segmentation anomalies in the cervical region of the spine (Sparrow et al., 2010). In 2013, a large consanguineous Arab family was reported to have seven SCDO4 cases. The affected individuals were reported to have consistent features of SCDO4. Three of the affected individuals were presented with dextrocardia along with situs inversus. Two of the affected individuals also exhibited secondary features such as neural tube defects (spina bifida occulta, thoracic myelomeningocele, and Chiari II) (Sparrow et al., 2013)*.*

The *HES7* gene (MIM608059; NM_001165967.2) covers 1674 bp of the genomic region, has four exons, and encodes *HES7* protein. An *HES7* protein is a transcription factor that belongs to the hairy and enhancer of split family of bHLH transcription factors (NP_001159439.1). To date, only seven disease-causing homozygous mutations have been identified in the *HES7* gene (HGMD, 2022). Only five mutations including four missense mutations and one small insertion have been associated with SCDO4 (Table 2).

Gene expression analysis of various transcription factors in an embryonic mouse brain have suggested the low level expression of *HES7* in the mid-brain and thalamus and thus, is limited to PSM (Gray et al., 2004). The expression of other members of the *HES* family (*HES*1, 3, 5, and 6) in the embryonic mouse brain has also been reported (Gray et al., 2004). Furthermore, severe neural tube defects and premature neurogenesis have been reported for *HES1*-null mutants (Ishibashi et al., 1995). Severe somite segmentation defects have been reported in homozygous *HES*7-null mouse embryos; laterality defects, however, have not been described (Bessho et al., 2001).

### 1.7 Spondylocostal dysostosis type 5 (SCDO5; *TBX6*)

SCDO5 was reported for the first time by Gucev et al. (2010) in a Macedonian descendant family segregating autosomal dominant spondylocostal dysostosis. The affected individuals had several phenotypes, such as disproportionate short trunks, short necks, mild scoliosis with hemi-vertebrae, and vertebral blocks. These affected individuals did not reveal any other abnormalities, dysmorphic features, or neurodevelopment issues. Gucev et al. (2010) identified a pathogenic heterozygous variant in the *TBX6* gene. In total, twenty-three individuals from the Chinese Han population with congenital scoliosis-related compound heterozygous mutations in the *TBX6* gene were identified. The affected individuals revealed hemi-vertebrae and other shared rib abnormalities (Wu et al., 2015). More recently (Lefebvre et al., 2017), identified homozygous disease-causing mutations in the *TBX6* gene justified the recessive inheritance of SCDO5. The affected individuals presented features such as disproportionate short stature, short necks, rib abnormalities, missing ribs, scoliosis, syringomyelia, and butterfly vertebrae.

The *TBX6* gene is located on chromosome 16p11.2 (NM_004608.3, NP_004599.2), and is composed of nine exons encoding a 463-amino acid protein, *TBX6*. *TBX6* belongs to a family that is phylogenetically conserved and shares a common DNA-binding domain, the T-box. The T-box genes encode transcription factors that play an important role in the regulation of developmental processes. The knockout studies in mice have suggested the role of this gene in the specification of paraxial mesoderm structures (Zhao et al., 2018). To date, only 66 disease-causing homozygous mutations have been identified in the *HES7* gene (HGMD, 2022). Only six mutations in the *TBX6* gene have been associated with SCDO5, including four missense mutations, one nonsense mutation, and one small deletion (Table 2). Other phenotypes associated with *TBX6* pathogenesis include scoliosis, Müllerian aplasia, Mayer–Rokitansky–Küster–Hauser syndrome, congenital anomalies of the kidney, tetralogy of Fallot, and autism.

The *TBX6*-null/mh mice exhibited vertebral malformations, similar to those in humans; the lower part of the spine is affected by the vertebral malformations (Liu et al., 2019).

### 1.8 Spondylocostal dysostosis type 6 (SCDO6; *RIPPLY2*)

SCDO6, an autosomal recessive disorder, is caused by homozygous variants in the *RIPPLY2* gene (NM_001009994.2), which is located on chromosome 6q14.2. SCDO6 exhibits severe phenotypes such as spinal canal stenosis, the descent of an occipital bone, absence of posterior elements of upper cervical vertebrae, hemi-vertebrae, butterfly vertebrae, cervical kyphosis, and thoracic scoliosis (Menger et al., 2021). McInerney-Leo et al. (2015) reported two affected individuals born to non-consanguineous parents having phenotypes such as affected posterior C1–C4 elements, butterfly vertebrae of T2–T7, cervical kyphosis, spinal canal stenosis, mild thoracic scoliosis, and hemi-vertebrae.

The *RIPPLY*2 gene consists of four exons (664 bp) that encode a 128-aa long nuclear protein, *RIPPLY2* (*RIPPLY* transcriptional repressor 2 protein), that belongs to a novel family of proteins required for vertebrate somitogenesis. The members of this family of proteins are characterized by having a tetrapeptide WRPW motif at N-terminal and a *RIPPLY* homology domain at C-terminal (composed of a Bowline-DSCR-Ledgerline conserved region) The *RIPPLY* family of proteins has been reported to be the transcriptional repressors as they negatively regulate the T-box proteins, including *TBX6*, coded by SCDO gene 10 (Kawamura et al., 2008). The transcriptional repression occurs by the interaction of *RIPPLY* proteins with the DNA-binding domain of T-box proteins, *via* their *RIPPLY* homology domain, and with the transcriptional co-repressor Groucho/TLE proteins *via* their WRPW motif (Kawamura et al., 2005). To date, only three disease-causing mutations have been identified in the *RIPPLY*2 gene, including one nonsense, one splice site mutation, and one small deletion (Table 2; HGMD, 2022).

Studies involving mice, which are kept homozygous null for *RIPPLY2*, have been reported to have severe vertebral and ribs malformations. Such mice also displayed defects in axial skeleton segmentations due to defective somitogenesis and finally, died in their prenatal stage. The vertebral defects that have been observed in *RIPPLY2*-null mice had a metameric pattern of vertebral bodies, intervertebral discs, and severely disrupted neural arches (Chan et al., 2007). Such conditions are similar to those of the mice, which are homozygous null for the autosomal recessive SCDO genes *DLL3*, *MESP2*, *LFNG*, and *HES7* (Sparrow et al., 2011).

### 1.9 Spondylocostal dysostosis type 7 (SCDO7; *DLL1*)

SCDO type 7 is a recently identified type of spondylocostal dysostosis inherited in an autosomal recessive manner. Barhoumi et al. (2019) reported two affected individuals (boys) showing features such as scoliosis, fused thoracic spines (T4–T5, T6–T8, and T11–T12), and multiple spine deformities. The whole-exome sequence analysis of these patients revealed a homozygous missense mutation (c.1534G>A; p. Gly512Arg) in exon 9 of the *DLL1* gene. In addition, 14 patients from unrelated families had neurodevelopmental disorders along with other brain abnormalities. The sequence analysis of these patients revealed the heterozygous mutations in the *DLL1* gene (Fischer-Zirnsak et al., 2019). All these reported patients had kyphosis/scoliosis, hyperextensible joints, hypotonia, and ataxia, as common features.

*DLL1*, located on chromosome 6q27, comprise a total of 11 exons (3174 bp) encoding a 723-amino acid long protein (NM_005618.4, NP_005609.3, 11). *DLL1* encodes for the protein Delta-like canonical Notch ligand 1 (*DLL1*). *DLL1* belongs to the Delta/Serrate/Jagged Family and is a human homolog of the *Drosophila* Delta ligand. It is important for hematopoiesis as it plays an important role in mediating cell fate decisions *via* cell-to-cell communication (Barhoumi et al., 2019). To date, 38 disease-causing mutations have been identified in the *DLL1* gene associated with different disorders such as neurodevelopmental disorder, autism, hearing loss, congenital heart disease, and cleft lip/or palate. However, only one mutation has been associated in *DLLI* causing vertebral malformations (SCDO7) (Barhoumi et al., 2019).

*DLL1* is composed of three domains, mainly intracellular, transmembrane, and an extracellular domain [containing eight epidermal growth factor-like (EGF-like) domains]. The mutation is mainly located in the eight EGF-like domains at a conserved nucleotide region. The mutation resulted in a change of amino acid at 512 position (from glycine to arginine), consequently affecting its binding properties with the target proteins.

Knockout mutations of *DLL1* in the mouse model generated a phenotype that differed significantly from the wild-type mouse in reference to the body size. The KO mouse had a small size and suffered from osteosclerosis because of the loss of function of osteoblasts and osteoclasts. According to the histopathological study, the affected mice had a reduced bone formation rate, decreased osteoblast surface area, and a compromised metabolic bone turnover (Muguruma et al., 2017).

### 1.10 Molecular genetics of SCDO and involvement of the Notch-signaling pathway

In humans, vertebrae are formed during embryogenesis from somites which are the embryonic segments produced from the presomitic mesoderm (PSM) in approx. 4–5 h. These somites are produced in a rhythmic sequence following an anterior to posterior direction. This periodic series of somite formation involves a molecular segmentation clock which basically operates through a Notch-signaling pathway (Wahi et al., 2016). Most of the genes belonging to the Notch-signaling pathway are involved in the somitogenesis and the molecular oscillation generated by them is synchronized with coupling genes of Wnt and FGF pathways in the PSM. These pathways are vital for normal vertebral development and somitogenesis as the coupled oscillations of the signaling genes under these signaling pathways govern the accurate segmentation in the process of somite formation. The mutations in these genes such as *LFNG*, *DLL3*, and *MESP2* have been shown to be associated with the abnormal molecular oscillations leading to the development of congenital vertebral malformations, that is, recessive spondylocostal dysostosis (SCDO) (Dequéant et al., 2006; Goldbeter and Pourquié, 2008).

The Notch-signaling pathway was identified for the first time in *Drosophila*. It is highly conserved through evolution from invertebrates to vertebrates (Bray, 2006). The Notch-signaling pathway is a very diverse and important pathway, which is known to interact with several other pathways such as hedgehog (Hh), fibroblast growth factor (FGF), Janus kinase/signal transducers, transcription activation (Jak/STAT), transforming growth factor-β (TGF-β), receptor tyrosine kinase (RTK), hypoxia pathways, and Wnt pathways (Gustafsson et al., 2005; Hurlbut et al., 2007). These signaling pathways have been extensively studied, and the future research might unravel the complex interactions and pathophysiology involved.

**FIGURE 2:**
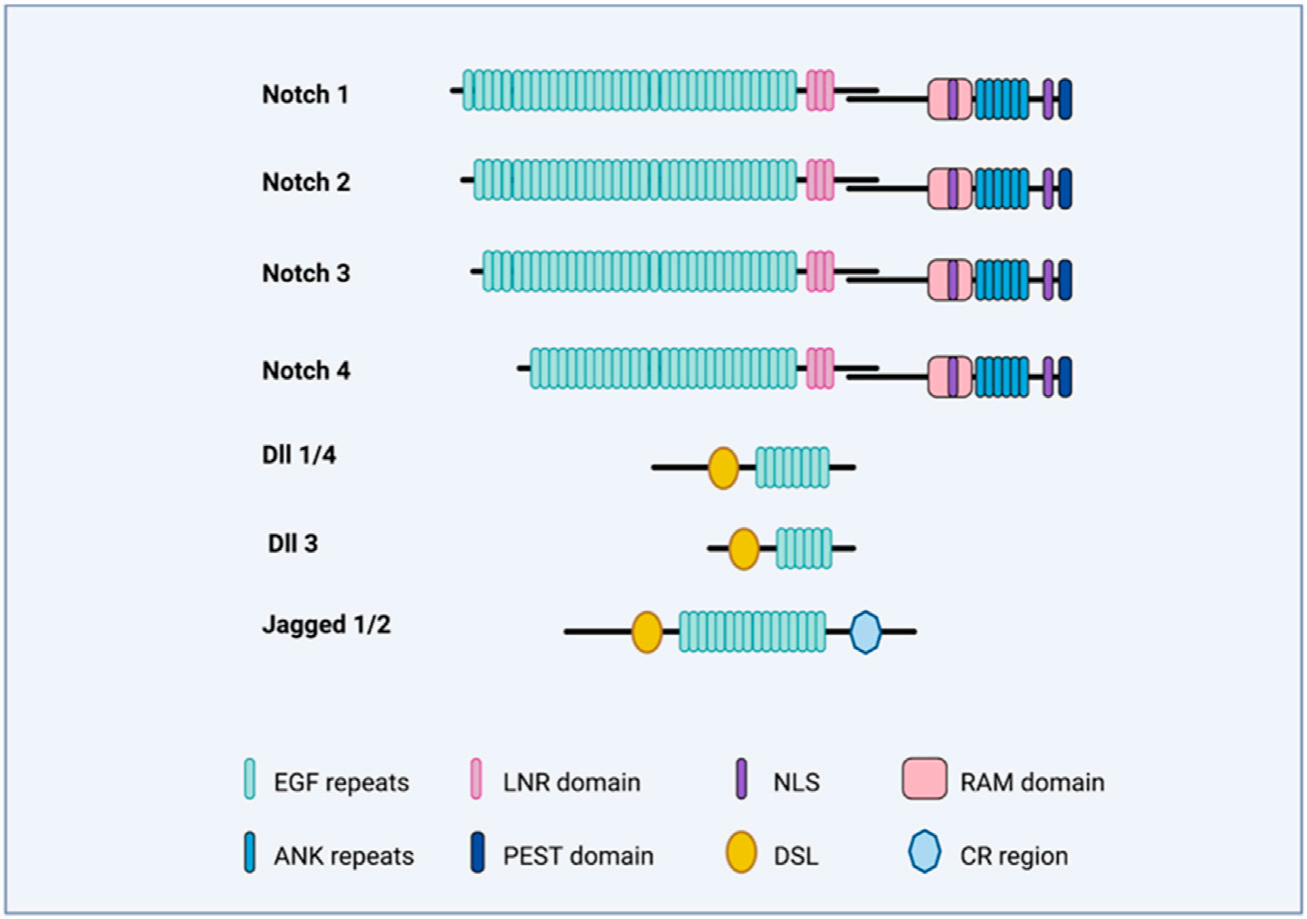
Notch-signaling illustration. The figure illustrates the domains of Notch receptors (Notch 1–4) and Notch ligands (*DLL1*,3,4; Jag1 and Jag2).

Notch receptors bind the Delta/Serrate/Lag2 ligands, also known as DSL ligands. In mammals, the DSL ligands are sub-divided into five types, which are grouped into two classes, that is, the Delta-like (homology with *Drosophila* Delta), which includes the *DLL1*, *DLL3*, and *DLL4*, as well as Serrate-like and the Serrate, which included *Jagged1* and *Jagged2* (Figure 2). Both the Delta-like and Serrate-like ligands have an extracellular domain having a signal peptide, an amino-terminal domain, a DSL domain, and several EGF-like repeats (Nye and Kopan, 1995).

In mammals, Notch receptors (Notch1–4) have four types (Figure 2), and it is structurally divided into an extracellular and intracellular domain. The extracellular domain (ECD) has EGF-like 29–36 repeats and three Lin-12/Notch (LIN) repeats. The EGF-like repeats binds the ligand, while the LIN repeats prevent ligand-independent signaling. The intracellular domain (ICD) contains the RBPj-associated molecule (RAM) domain, and a six-ankyrin (ANK) repeats domain. Both of these are protein-interacting domains. Apart from these, the intracellular domain also contains a transactivation domain (TAD), a PEST sequence, and nuclear localization signals (NLSs).

**FIGURE 3:**
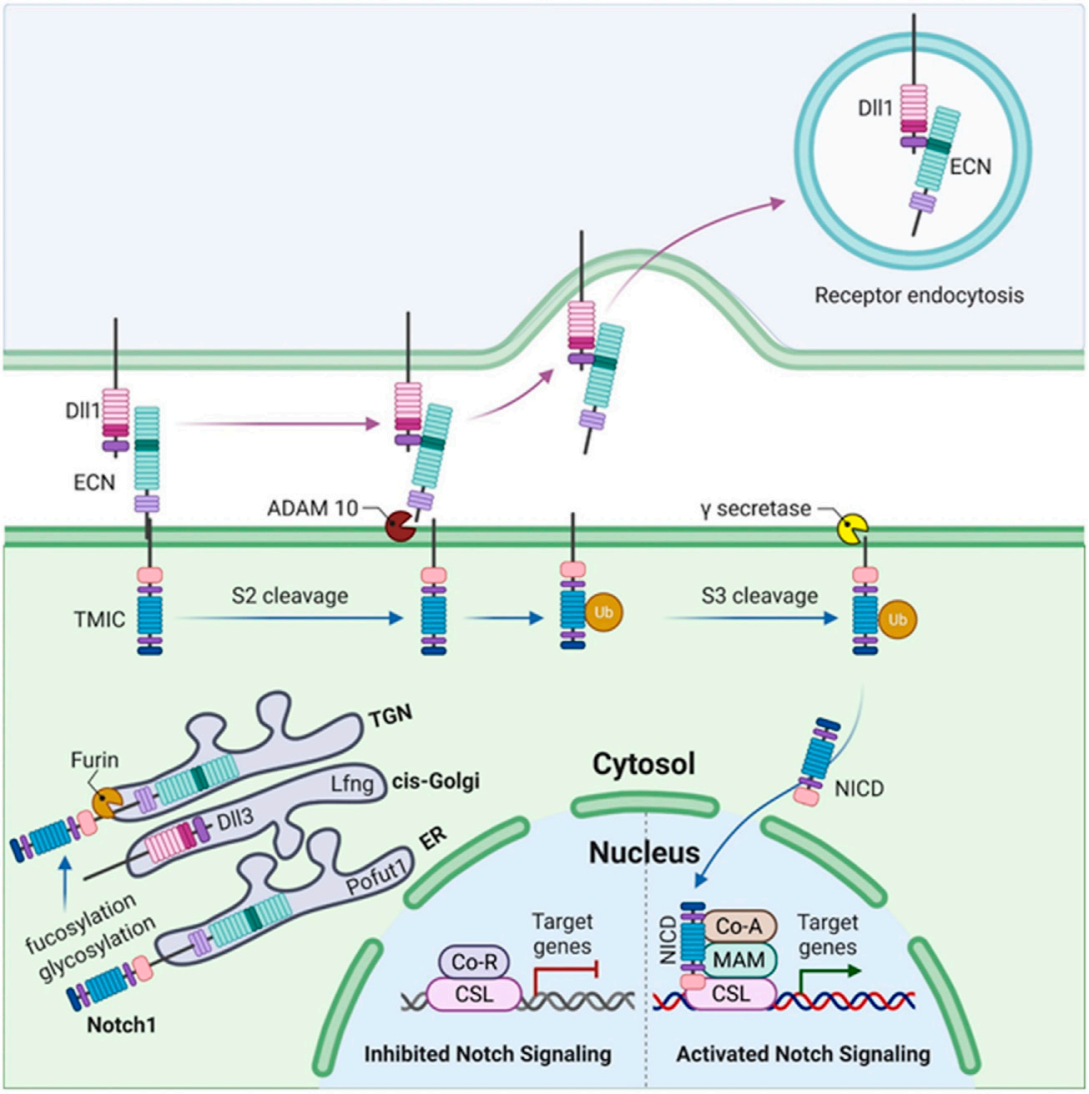
Somitogenesis in mammals illustrating Notch1 signaling. The figure illustrates the synthesis of Notch1 as a single polypeptide. O-fucosylation of specific EGF-like repeats occurs in the endoplasmic reticulum (ER) by O-fucosyltransferase 1 (Pofut1), followed by the elongation of these O-fucosylated EGF-like repeats (upon addition of GlcNAc by lunatic fringe, Lfng) as the Notch1 polypeptide passes through the cis-Golgi. S1 cleavage occurs in the trans-Golgi network (TGN) by a furin-like convertase. The Notch1 heterodimer (composed of an N-terminal extracellular truncation, ECN, and a C-terminal transmembrane and intracellular domain, TMIC) moves toward the cell surface where it binds *DLL1 via* ECN in trans. This binding leads to the activation of S2 cleavage by the disintegrin and metallopeptidase domain (ADAM) proteases, ADAM10 or ADAM17. This liberates the Notch1 ECN which then undergoes ubiquitylation (Ub) and is endocytosed. S3 cleavage of Notch1 ECN occurs in the transmembrane domain *via* γ-secretase which releases the Notch1 intracellular domain (NICD). NICD binds CSL which is a DNA-binding protein. Upon binding to CSL, co-repressor (CoR) proteins and histone deacetylases (HDACs) are released while the binding of coactivators (CoAs) occurs simultaneously which results in the activation of Notch signaling, leading to the transcription of target genes such as *MESP2*, *LFNG*, and *HES7*.

The Notch-signaling pathway is very simple, as the intracellular domain (ICD) of the Notch receptor lacks the second messengers and is thus directly involved in the target gene transcription (Figure 3). However, Notch-signaling modifiers have been involved in the complex signaling specificity (Bray, 2006). Once synthesized as a single polypeptide, Notch protein travels through the Golgi apparatus where it may be further modified (Panin et al., 1997) and is cleaved by a furin-like convertase *via* S1 cleavage in the trans-Golgi network convertase (Logeat et al., 1998). The associated specific epidermal growth factor (EGF)-like repeats are modified in the endoplasmic reticulum (ER) by O-fucosylation *via* O-fucosyltransferase 1 (Pofut1).

The C-terminal transmembrane, N-terminal extracellular truncation (ECN), and intracellular domain (TMIC) of the Notch receptor fragments form a heterodimer that proceeds toward the cell surface (Rand et al., 2000; Sanchez-Irizarry et al., 2004). Once it has reached the cell surface, it is cleaved by disintegrin (S2 cleavage) and proteases (metalloprotease domain, ADAM10 and ADAM17) due to the interaction of the ECN with the respective ligand (in the trans), resulting in the separation of Notch heterodimer separation through transendocytosis of the ECN into the signal-sending cell (Pan and Rubin, 1997; Brou et al., 2000; Hartmann et al., 2002). The Notch-signaling pathway in Figure 3 summarizes the function and involvement of each gene.

### 1.11 Diagnosis and genetic counseling

SCDO is mostly diagnosed by the radiological features in association with NGS (WES/WGS). The identification of the culprit gene defines the type of SCDO. It is characterized into different subtypes as a result of homozygous mutations in several genes causing autosomal recessive SCDO, such as *DLL3*, *DLL1*, *LFNG*, *RIPPLY2*, *MESP2*, *HES7*, and *TBX6*. Once the mutation in the specific gene is identified, genetic counseling and family carrier testing can be performed. Carrier testing will help to reduce the occurrence of diseases in coming generations (Umair et al., 2017).

## 2 Discussion

Genetic deformities of the skeletal system are characterized by malformation, inconsistent growth, and distortion of individual bones or groups of bones, which can cause syndromic and non-syndromic forms (Umair et al., 2019). Genetic skeletal deformities can occur due to disruption in the intricate processes of skeletal development, growth, and homeostasis. The inheritance pattern of genetic skeletal deformities can be either autosomal (dominant or recessive) or X-linked (dominant or recessive) (Umair et al., 2018b; Umair and Hayat, 2020; Ahmad et al., 2022). Similarly, spondylocostal dysostosis is a severe form of skeletal deformity that leads to severe ribs and vertebrae malformations. With the advent of NGS technology, molecular diagnosis of rare skeletal deformities such as spondylocostal dysostosis is now cost effective and quick (Umair et al., 2018a). Screening the associated seven genes in patients suffering from disorders such as spondylocostal dysostosis will enable the researchers and clinicians to treat the patient at an early stage. In addition, techniques such as Noninvasive prenatal testing (NIPT), Pre-implantation genetic testing for aneuploidy (PGT-A) and Pre-implantation genetic testing for monogenic disorders (PGT-M) have been employed to reduce the occurrence of severe genetic disorders (Alyafee et al., 2021a; Alyafee et al., 2021b; Alyafee et al., 2022). Therapeutic interventions are only possible if we know the exact molecular origin of a disease that might help the researchers to identify the exact biomarkers. In such a scenario, knowledge about the molecular etiology and pathophysiology of such a rare genetic disease is a must to implement and draw future therapeutic interventions.

The Notch-signaling pathway plays a major role in numerous cell developmental processes, the major one being the skeletal system development and bone homeostasis. The Notch-signaling system utilizes Notch receptors as a key component in the regulation of cell differentiation and function. The four Notch receptors carry out cell-specific functions in the skeletal system and any mutations in them cause alterations in the Notch signaling. Several skeletal malignancies and development of various congenital bone disorders have been reported to be associated with the variations in the Notch-signaling pathway (Canalis, 2018).

The Notch-signaling pathway is an absolute requirement for normal somitogenesis in vertebrates which has been confirmed by mouse models and cell culture studies. The somite boundary formation and its patterning largely depend on the Notch1 activity. The Notch pathway genes are largely involved in the rostral-caudal patterning, synchronized oscillations in the segmentation clock in PSM, and the segmental border formation. Disease causing genetic mutations encoding the protein involved in these essential processes has been reported to be the source of severe vertebral deformities in humans Notch pathway components (Dunwoodie SL., 2009).

Investigation of the genetic causes of abnormal vertebral segmentation (AVS), using mouse models, has reported certain genes that are important for normal vertebral development as they are the activators of Notch, FGF, and Wnt signaling pathways. These signaling pathways are pivotal for normal somite production and bones development in vertebrates. Mutations in these genes, that is, *DLL3*, *MESP2*, *LFNG*, and *HES7*, cause congenital AVS disorder, SCDO. *DLL3* and *DLL1* are worth mentioning as these genes serve as the DSL ligands of Notch and are likely to inhibit the pathway by acting as an inhibitor of signaling (Dunwoodie S. L., 2009).

Disease-causing variants in Notch-signaling pathway members cause different types of developmental disorders that affect different parts of the body, including the skeleton, heart, liver, kidneys, eye, face, and vasculature. Notch-associated disorders include different types of severe disorders such as Alagille syndrome caused by mutations in both ligand JAG1 and receptor Notch2 and autosomal recessive spondylocostal dysostosis, caused by mutations in ligand *DLL3*, and many other members of the Notch-signaling pathway (Penton et al., 2012). Similarly, the other genes associated with recessive spondylocostal dysostosis (*MESP*2, *LFNG*, and *HES*7) have been associated with regulating Notch-signaling activity during somitogenesis (Penton et al., 2012).

**FIGURE 4:**
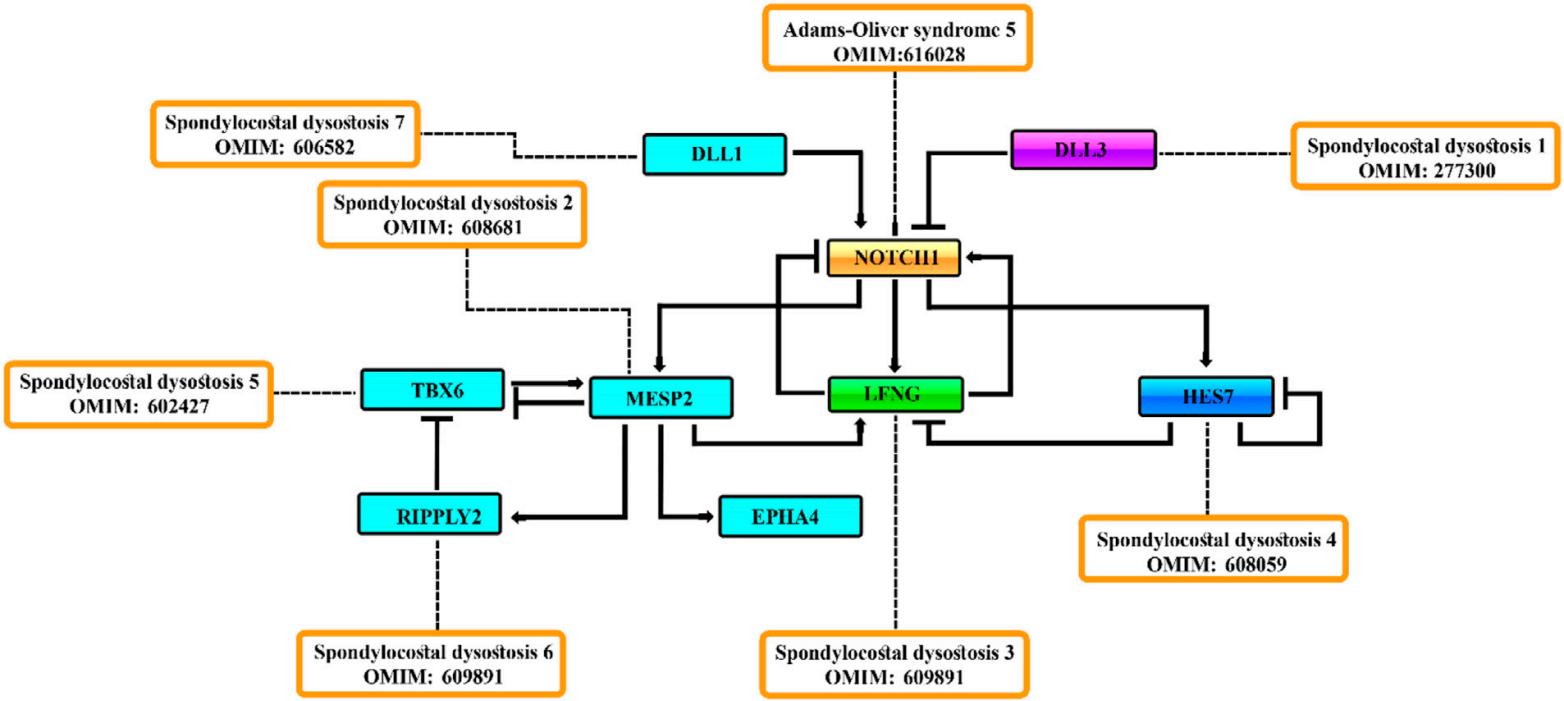
Negative feedback [act as repressors] loop consisting of the *MESP2*, *LFNG*, *HES7*, and *DLL3* genes regulate the Notch-signaling activity during somitogenesis.

It has been observed that *DLL3* interacts with full-length Notch1 in the late endocytic compartment and that high levels of *DLL3* expression are correlated with low levels of Notch1, suggesting that *DLL3* targets Notch1 for lysosomal degradation (Chapman et al., 2010). The *LFNG* is an N-acetylglucosaminyltransferase that adds N-acetylglucosamine to fucose residues on Notch receptors and acts to suppress Notch signaling during somitogenesis in mice (Sparrow et al., 2006). *HES7* acts to downregulate *LFNG* expression. *LFNG*, *DLL3*, and *MESP2* mutant embryos display a broadening of Notch-signaling activity during somitogenesis, detected by antibodies to NICD (Chapman et al., 2010; Feller et al., 2008) (Figures 3, 4). *DLL3* is predicted to participate in lysosomal degradation of Notch1, *MESP2* destabilizes MAML, and *LFNG* down-regulates Notch signaling immediately posterior to the forming somite (Chapman et al., 2010; Sasaki et al., 2011) (Figures 2, 4). Not surprisingly, ubiquitous activation of Notch in the presomitic mesoderm results in abnormal somite formation (Feller et al., 2008).

## 3 Conclusion

In conclusion, proper somite formation is dependent on the Notch-signaling pathway which regulates the skeletal homeostasis. SCDO is an abnormal congenital disorder of vertebral and rib deformation which occurs due to the mutations of the genetic components of the Notch-signaling pathway. SCDO is diagnosed based on characteristic symptoms, family history, an extensive clinical evaluation (radiographs of the spine), and molecular diagnosis (WGS/WES). No specific treatment is available; however, surgery can be performed to repair an inguinal hernia and scoliosis, and antibiotics can be suggested for recurrent respiratory infections. As a rare genetic disorder, the exact incidence or prevalence of SCDO is unknown, and a proper genetic and molecular analysis is required to identify the culprit gene, which might help in proper diagnosis and timely treatments.
